# Adhesion molecules and cerebral microvascular hemodynamic abnormalities in sickle cell disease

**DOI:** 10.3389/fneur.2022.976063

**Published:** 2022-12-07

**Authors:** Noor Mary Abi Rached, Oluwabukola T. Gbotosho, David R. Archer, Jayre A. Jones, Morgan S. Sterling, Hyacinth I. Hyacinth

**Affiliations:** ^1^Neuroscience and Behavioral Biology Undergraduate Program, Emory University, Atlanta, GA, United States; ^2^Department of Neurology and Rehabilitation Medicine, University of Cincinnati College of Medicine, Cincinnati, OH, United States; ^3^Aflac Cancer and Blood Disorders Center, Emory University Department of Pediatrics and Children's Healthcare of Atlanta, Atlanta, GA, United States

**Keywords:** sickle cell disease, adhesion molecules, microvascular hemodynamics, cerebral microinfarct, two-photon microscope

## Abstract

Cerebrovascular abnormalities are a common feature of sickle cell disease that may be associated with risk of vaso-occlusive pain crises, microinfarcts, and cognitive impairment. An activated endothelium and adhesion factors, VCAM-1 and P-selectin, are implicated in sickle cell vasculopathy, including abnormal hemodynamics and leukocyte adherence. This study examined the association between cerebral expression of these adhesion factors and cortical microvascular blood flow dynamics by using *in-vivo* two-photon microscopy. We also examined the impact of blood transfusion treatment on these markers of vasculopathy. Results showed that sickle cell mice had significantly higher maximum red blood cell (RBC) velocity (6.80 ± 0.25 mm/sec, *p* ≤ 0.01 vs. 5.35 ± 0.35 mm/sec) and more frequent blood flow reversals (18.04 ± 0.95 /min, *p* ≤ 0.01 vs. 13.59 ± 1.40 /min) in the cortical microvasculature compared to controls. In addition, sickle cell mice had a 2.6-fold (RFU/mm^2^) increase in expression of VCAM-1 and 17-fold (RFU/mm^2^) increase in expression of P-selectin compared to controls. This was accompanied by an increased frequency in leukocyte adherence (4.83 ± 0.57 /100 μm/min vs. 2.26 ± 0.37 /100 μm/min, *p* ≤ 0.001). We also found that microinfarcts identified in sickle cell mice were 50% larger than in controls. After blood transfusion, many of these parameters improved, as results demonstrated that sickle cell mice had a lower post-transfusion maximum RBC velocity (8.30 ± 0.98 mm/sec vs. 11.29 ± 0.95 mm/sec), lower frequency of blood flow reversals (12.80 ± 2.76 /min vs. 27.75 ± 2.09 /min), and fewer instances of leukocyte adherence compared to their pre-transfusion imaging time point (1.35 ± 0.32 /100 μm/min vs. 3.46 ± 0.58 /100 μm/min). Additionally, we found that blood transfusion was associated with lower expression of adhesion factors. Our results suggest that blood transfusion and adhesion factors, VCAM-1 and P-selectin, are potential therapeutic targets for addressing cerebrovascular pathology, such as vaso-occlusion, in sickle cell disease.

## Introduction

Sickle cell disease (SCD) is caused by a single point mutation in the beta-globin gene, resulting in the substitution of valine for glutamic acid in the resulting β-globin peptide. In turn, this leads to polymerization of deoxyhemoglobin, forming sickled erythrocytes (1). SCD impacts an estimated 100,000 individuals in the United States, and the incidence of SCD among African Americans is approximately 1 in 360 newborns (2, 3). SCD involves clinical complications that impact multiple organ systems, with pain being the most frequent and results from vaso-occlusion. As a common symptom of SCD, vaso-occlusive pain crises result from sickle shaped erythrocytes and leukocytes blocking blood flow, particularly in small vessels, resulting in ischemia of organs and thus, pain (4, 5). Additionally, these vaso-occlusive events (VOEs) can be triggered by processes such as inflammation, thrombosis, increased aggregation of cells, and adhesion of blood cells to the vascular endothelium, ultimately leading to blockages that deprive the tissues of nutrients and oxygen (6–9). This results in tissue death and infarction in several organ systems including the spleen, liver, kidney, and lungs (4, 10). Another possible consequence of VOEs is silent cerebral infarction (SCI) or cerebral microinfarctions, which are small ischemic lesions that may occur without overt neurological symptoms. Studies have shown that SCIs or cerebral microinfarcts may be linked to the development of cognitive decline, and are associated with vascular and hemodynamic abnormalities such as cerebral macro and/or micro-vasculopathy, hypoperfusion, or obstructions to blood flow (11–13). Magnetic resonance imaging (MRI) studies from clinical cohorts in the Cooperative Study of Sickle Cell Disease showed that children with silent cerebral microinfarcts also have a higher risk of stroke (14, 15). Further studies from this cohort showed that school-aged children with SCD and silent infarcts experienced difficulties with neuropsychological functions (16). In mouse models, a recent study from our laboratory showed that sickle cell (SS) mice had 2.5 times more cortical microinfarcts than controls. Additionally, these mice had significantly higher prevalence of evidence of spontaneous cerebral vasculopathies, such as higher red blood cell (RBC) velocity, more tortuous capillary vessels, and more frequent capillary occlusive events (12).

Vaso-occlusive events occur more frequently in the low flow “micro” vessels such as, the pre-capillary arterioles, capillaries, and post-capillary venules (17, 18). Studies in non-sickle cell mouse models showed that blockage or experimental occlusion of the principal (penetrating) cortical venules (PCV) lead to stagnant flow in the upstream arterioles. This impairs blood flow into the cortex, thus, highlighting the importance of the entire cerebral microvascular tree, in the etiology of cerebral microinfarcts (11). However, it is important to note that SS RBCs typically pass through the capillary bed prior to hemoglobin polymerization, suggesting that additional factors may also be involved in the pathology of VOEs in SCD (19, 20). For instance, endothelial activation has been shown to play a role in the pathogenesis of VOEs in SCD (8, 18, 21). This is shown by the demonstration of significantly higher levels of circulating endothelial cells in patients with SCD compared with matched controls (21). *In vitro* studies also demonstrated the capacity for the more rigid sickle erythrocytes to mechanically activate endothelial cells, leading to an increase in expression of cellular adhesion molecules (markers of endothelial activation) which in turn propagates further adhesion and vaso-occlusion in a vicious cycle (8, 22, 23). The role of these cellular adhesion molecules [such as Intercellular Adhesion Molecule 1 (ICAM-1), P- and E-selectins and vascular cell adhesion molecule 1 (VCAM-1)] and thus endothelial activation in VOEs is further supported by a recent report of higher levels of E-selectin, VCAM-1, and ICAM-1 in SCD patients compared to controls (18). One study demonstrated significantly higher levels of these molecules, suggesting endothelial activation among SCD patients presenting with complications and even higher levels among those presenting with an active vaso-occlusive pain crises, compared to steady state (24). It is already well demonstrated that individuals with SCD have elevated leukocyte counts, which in the setting of increased VCAM-1 expression, results in increased endothelial interaction and thus arrest (25, 26). Our lab reported a strong relationship between serum soluble VCAM-1 (sVCAM-1), P-selectin and ICAM-1 levels, and risk of stroke in patients (children) with SCD. In the same study, we also demonstrated that lower levels of these cell adhesion markers were associated with stroke free survival as well as use of blood transfusion therapy for stroke prevention in patients with SCD (27). A paradigm proposed by Frenette et al. (28) suggests a multi-step model of vaso-occlusion whereby sickle cells induce endothelial activation, creating an environment where adherent leukocytes can interact with both RBCs and the endothelium to hinder blood flow, and subsequently create blockages (28, 29). In addition, it has been documented that among patients with sickle cell disease, those with higher cerebral blood flow as a compensatory mechanism for lack of brain oxygenation, performed more poorly on tests of cognitive function (30). This highlights the importance of exploring how adhesion factors may relate to this abnormal blood flow in SCD. Taken together we reasoned that the level of expression (or deposition) of cellular adhesion molecules in the cerebral microvascular endothelium will play a role in cerebral microvascular hemodynamics and could be a physiological mechanism for the cortical microinfarct or SCIs observed in mouse models of SCD and adults/children with SCD respectively (12, 25, 31, 32).

Vascular cell adhesion molecule-1 (i.e., VCAM-1), is expressed on blood vessels (endothelial cells) after activation by chemical (such as cytokines and chemokines) and/or mechanical stimulation, resulting in cytokine release. It is involved in adhesion of lymphocytes, monocytes, eosinophils, and basophils to the endothelium (33). According to Stuart and Setty (34), in a state of hypoxia, sickled red blood cells adhere to endothelial VCAM-1 using the very late activation antigen-4 (VLA4) ligand (34). P-selectin has also been implicated in the pathophysiology of SCD and is currently the target of a newly developed anti-vaso-occlusive crisis (VOC) drug (35–42). The chronic inflammatory milieu of SCD results in a persistently elevated serum level as well as endothelial expression of P-selectin, which is necessary for the initial binding of leukocytes to the vascular endothelium (43, 44). However, in the setting of SCD, P- selectin expression is likely to result in disruption of normal hemodynamics from excess/aberrant leukocyte-endothelial interaction (45, 46). While studies have shown that excessive endothelial expression of VCAM-1 and P-selectin results in VOEs and therefore pain in the periphery, the impact of such excess adhesion molecule expression on leukocyte-endothelial interaction and therefore cerebral microvascular hemodynamics is not known. Our study investigated this relationship given its potential implication for cerebral/cortical infarction and therefore neurological complications such as cognitive impairment. We also examined the relationship between the deposition (expression) of the two most well documented cellular adhesion molecules, VCAM-1, and P-selectin, associated with SCD complications and cerebral microvascular hemodynamics. Finally, we examined the impact of packed red blood cell transfusion on these adhesion molecules and therefore cerebral hemodynamics.

## Methods

### Animal preparation

**Figure 1:**
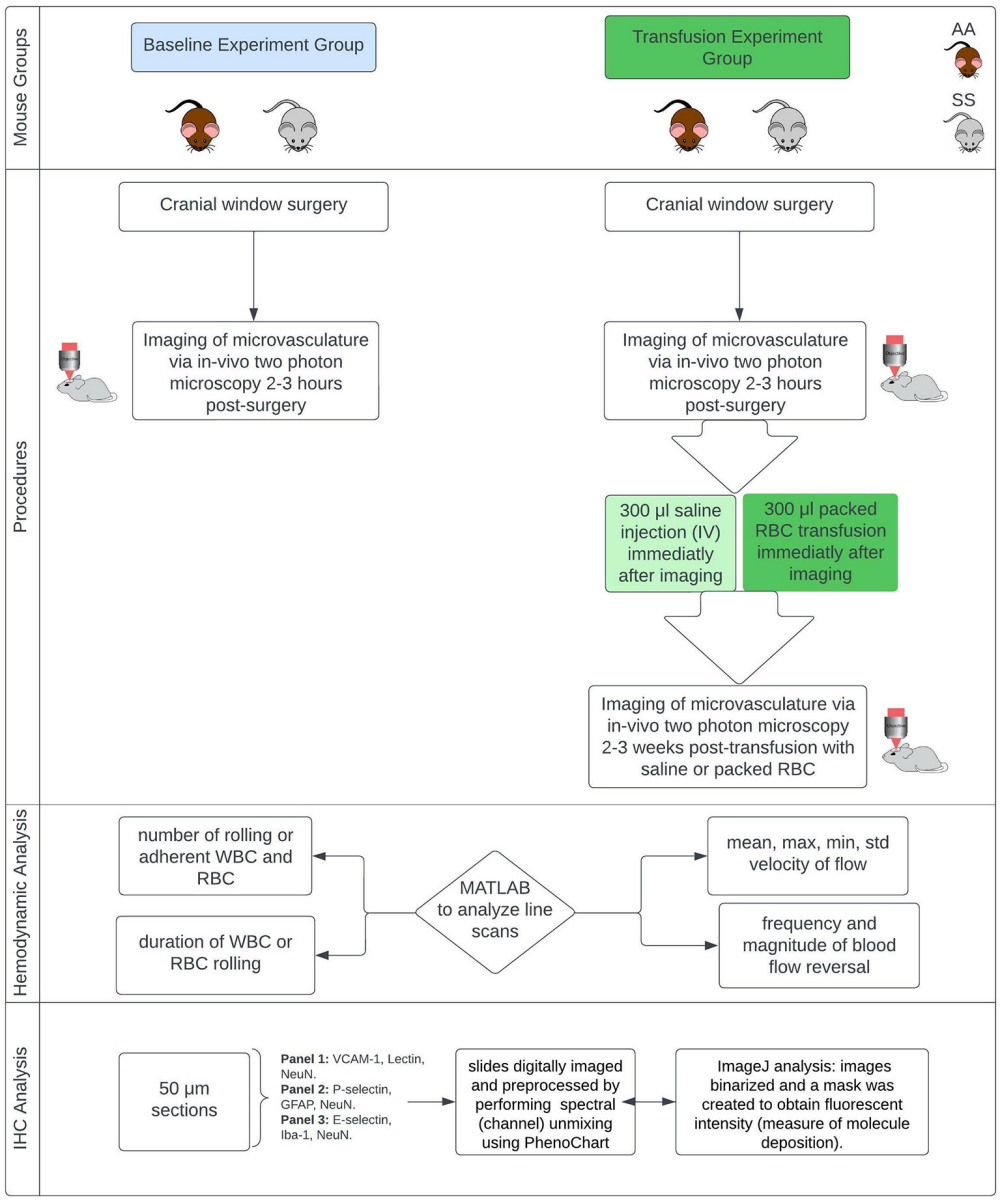
Overview of methods. Townes mouse model, a humanized sickle cell (with HbSS) and control (HbAA) mouse model (12–13 months). IHC, immunohistochemistry.

The Institutional Animal Care and Use Committees (IACUC) of Emory University and the Medical University of South Carolina approved this study, and all research was conducted in accordance with the National Research Council and National Institutes of Health *Guide for the Care and Use of Laboratory Animals 8th Edition* (47). Figure 1 provides an overview of the experiments.

This study used the Townes mouse model, a humanized sickle cell mouse model (with HbSS) and corresponding humanized control group (with HbAA). Mice are male and ~13 months old at the time of starting the experiments. They were divided into two main groups. Group one was used to examine changes in microvascular hemodynamics while group two was used to examine the impact of red blood cell transfusion on microvascular hemodynamics. Blood transfusion therapy is a primary treatment option for primary and secondary stroke prevention for children and adults with SCD and has been shown to reduce hemoglobin S concentration as well as reducing the risk of stroke and silent cerebral infarct (cerebral microinfarcts) (48–50).

In our study, all mice in both the baseline group and the transfusion experiment group underwent implantation of a cranial window (placed over the somatosensory cortex) under anesthesia to obtain optical access to the intracranial space. The procedures for anesthetizing mice and performing the cranial window surgery have been described in our prior publications (12, 51). Two to three hours following surgery, the mice in the blood transfusion group underwent pre-transfusion two-photon laser scanning microscopy (TPLSM) imaging. Afterwards (i.e., immediately following imaging and prior to recovery from anesthesia), sickle cell mice received blood transfusions with 300 μl of packed red blood cells from humanized Townes HbAA mice while control (HbAA) mice received 300 μl intravenous (IV) saline. All infusions were performed slowly over 1 min. The goal of the packed RBC transfusion was to raise the hemoglobin level by at least 1g/dL. Mice in the baseline group underwent post-surgery imaging, but they did not receive any transfusion fusions and were sacrificed immediately following the two-photon (2 Photon) imaging. The mice that received blood or saline infusion, underwent a second 2 Photon imaging 2–3 weeks after the infusion. It is important to note that, in the transfusion experiment each group of mice served as their own treatment control, thus the pre and post transfusion imaging represent the same group of mice at different time points. The AA mice also served an additional purpose of being a control for the impact of handling as well as to enable us to show that any positive effect of packed RBC transfusion observed in the HbSS mice, is not due to the passage of time and thus resolution of the initial stress from surgery.

### *In vivo* imaging procedure

To examine hemodynamic parameters, *in-vivo* images of cortical capillaries, precapillary arterioles, and post capillary venules (low flow cortical microvascular hemodynamics) were obtained using 2 Photon microscopy based on the schedule described above. Imaging was performed with a Sutter Moveable Objective Microscope (MOM) and a Coherent Ultra II Ti: Sapphire laser source. Methods for animal preparation during imaging and for measurement of cerebral hemodynamic and microvascular parameters *via in vivo* 2 Photon blood flow imaging have been previously described and published (12, 52, 53). about 2–5 min prior to commencing imaging, fluoresceine conjugated dextran (FITC-dextran 2kD to label the plasma) and Rhodamine 6G (to label leukocytes) are administered to the mice mouse IV, in that order.

### Hemodynamic analysis

To examine hemodynamic parameters, we used custom MATLAB codes to analyze line scans of cerebral capillary, pre-capillary and post-capillary blood vessel images acquired using 2 Photon microscopy from SS and control (AA) mice. Using the MATLAB scripts, we were able to determine the following: mean and standard deviation of RBC flow velocity, maximum and minimum velocity of RBC flow, frequency (per minute) and magnitude of microvascular RBC/blood flow reversal and leukocyte (WBC) rolling on the endothelium. Microvascular RBC or blood flow reversal is a change in the original direction of blood flow relative to the direction of the line scan and could be multiple transient changes or a single change that last the duration of the line scan. A rolling or adherent WBC is defined as WBC stagnation lasting two or more seconds. We normalized the number of leukocyte rolling or leukocyte adherence events to a fixed vessel segment (100 μm) per unit time (1 min). This is to ensure reproducibility of our findings (54). All image analysis was performed by members of our laboratory who were blinded to the genotype or transfusion status of the mice.

### Immunohistochemistry

At the end of 2 Photon imaging, the mice were sacrificed, and the brain extracted for immunohistochemistry (IHC) analysis to examine the microvascular deposition of adhesion factors, possible presence and size (area) of cerebral (cortical) microinfarcts, and possible presence of gliosis at the site of infarcts. The immunohistochemical protocol/approach has been previously described and published (12). For the baseline mice, the IHC was performed immediately following the imaging. For the mice that were transfused either with saline (HbAA) or packed RBC (HbSS), all IHC was performed after the post-transfusion 2 Photon imaging, and there was no pre-transfusion IHC analysis. We used the following primary antibody combinations to label 50-micron brain tissue sections from SS and AA mice. Panel 1 contained VCAM-1, Lectin (to localize and stain the vasculature), and NeuN (to localize and stain neuronal nuclei). Panel 2 contained P-selectin, GFAP (to localize and stain reactive astrocytes), and NeuN. Panel 3 contained E-selectin, Iba-1 (to localize and stain microglia), and NeuN. These slides are digitally imaged using a PerkinElmer digital slide scanner (Akoya Biosciences), and then images were preprocessed by performing spectral (channel) unmixing using PhenoChart (Akoya Biosciences). IHC Images were analyzed using ImageJ, with standard parameters for each fluorophore. Briefly, after spectral unmixing, the images from each fluorescent channel were transferred to image J, where they were binarized and a mask was created. The mask was then applied to the source images to obtain fluorescent intensity as an indication of the level of deposition (expression) of the molecule e.g., VCAM-1 of interest. The threshold values for creating the masks as well as analysis parameters were kept constant between images and between genotype (i.e., HbSS and HbAA) mice for each fluorophore/fluorescent channel and their adhesion molecule of interest. The resulting intensity was then normalized to a unit (mm^2^) image size and expressed in relative fluorescence unit (RFU) per mm^2^.

### Statistical analysis

We performed data analysis for comparison between sickle cell and control mice using GraphPad Prism software (GraphPad Software Inc, La Jolla, CA). We checked our data for normality using the Shapiro-Wilk test, and then we used the Welch corrected *t*-test for comparison of differences between sickle cell and control mice because of the heteroscedasticity in our data based on Levene's *F*-test for equality of variance. Our study minimum sample size was 3 mice per genotype group and was based on our prior experiments and publications using this mouse model (55–57). Quantitative results are presented using bar plots with means and standard error of means (SEM), comparing sickle to control mice and with a *p*-value of < 0.05 considered statistically significant. Qualitative data are presented as representative array of histochemical images.

## Results

### Cerebral hemodynamic properties at baseline for sickle cell mice compared to age-matched controls

**Figure 2:**
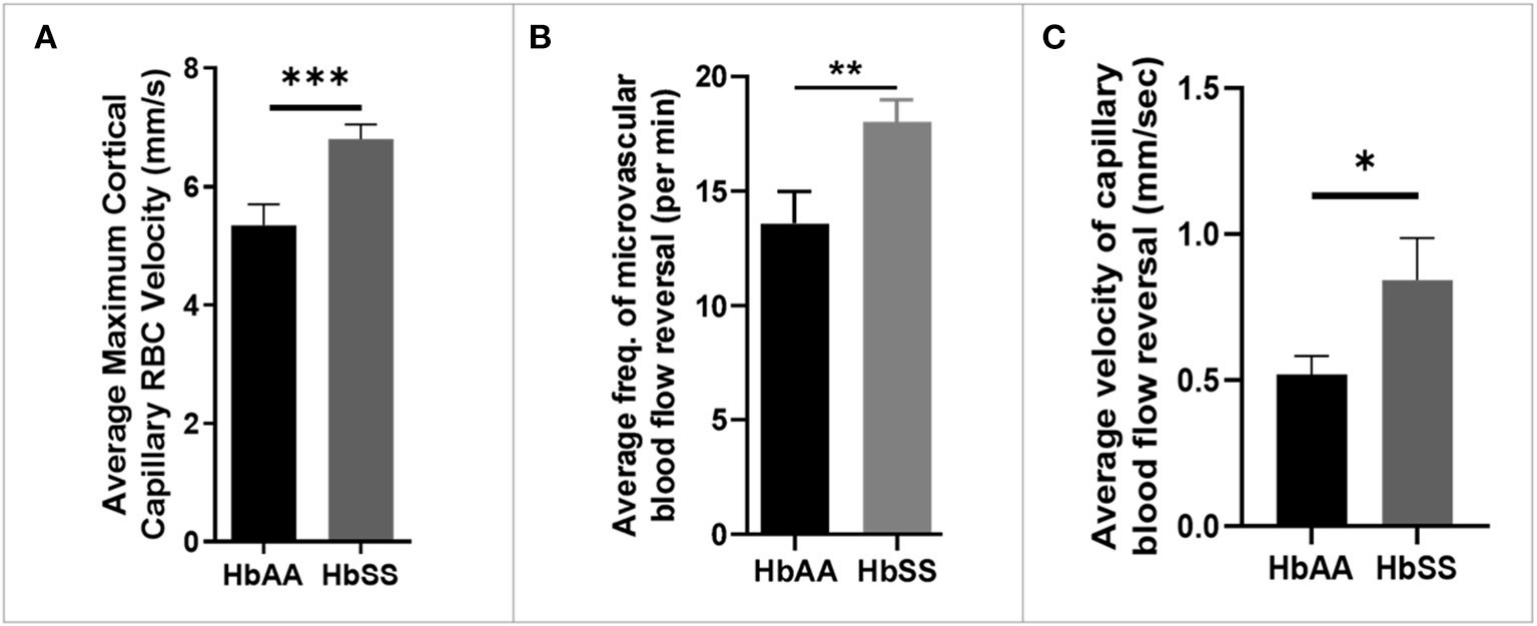
Altered capillary hemodynamics in 13-month-old sickle cell mice compared to age-matched controls. **(A)** average maximum capillary RBC velocity in millimeters per second. **(B)** average frequency of capillary blood flow reversal per minute **(C)** average velocity of capillary blood flow reversal in millimeters per second (AA: *n* = 5, average of ~194 vessel segments; SS: *n* = 7, average of ~390 vessel segments). Error bars are standard error of means (SEM). *p ≤ 0.05; **p ≤ 0.01; ***p ≤ 0.001. Mean comparisons done using Welch's corrected *t*-test. Results here are only from the mice in the baseline group.

Analysis of the 2 Photon microscopy imaging data for cerebral microvascular hemodynamic measurements revealed that sickle cell mice had significantly higher maximum RBC velocity (6.80 ± 0.25 mm/sec vs. 5.35 ± 0.35 mm/sec, *p* = 0.0009) compared to age-matched controls (Figure 2A). In addition, we noted that that sickle mice have a higher frequency of cerebral microvascular blood flow reversal (18.04/min vs. 13.59/min, *p* = 0.008) compared to controls (Figure 2B). Further analysis revealed that the velocity of blood flow reversal was also significantly higher among sickle cell mice (0.84 ± 0.14 mm/sec vs. 0.52 ± 0.06 mm/sec, *p* = 0.03) compared to controls (Figure 2C). Also, blood flow reversal parameters are used to identify how often and at what velocity blood flow in a vessel is disturbed (58–60). This suggests that blood flow is abnormal in sickle cell mice, and this may be related to VOEs.

### Sickle cell mice have elevated expression/deposition of endothelial adhesion factors (VCAM-1 and P-selectin) compared to controls

**Figure 3:**
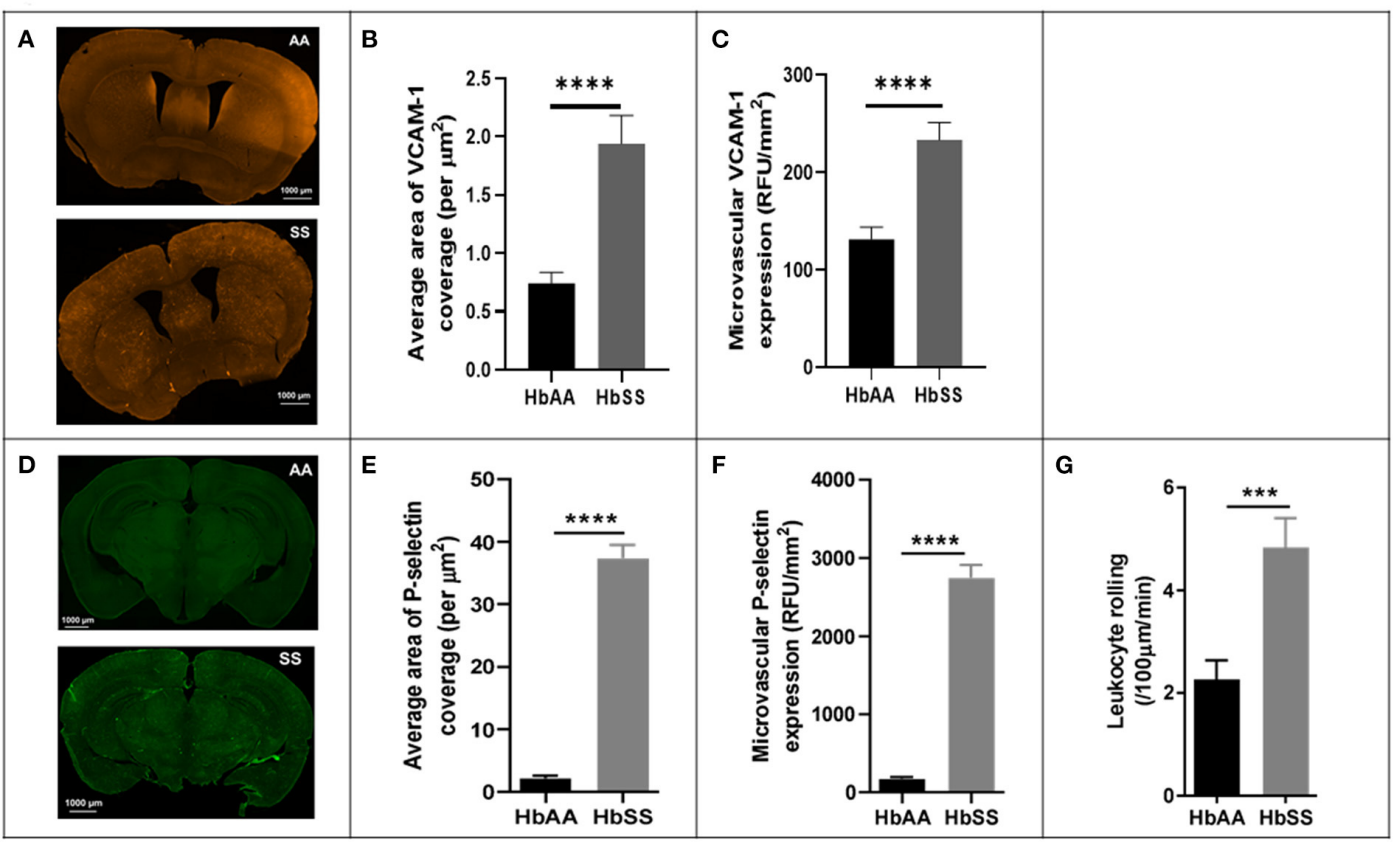
Adhesion factor expression and the rate of leukocyte adherence are elevated in the brains of sickle cell mice. Fluorophore of interest was isolated to show fluorescent areas representing VCAM-1 deposition along the microvasculature. **(A)** Representative image of stained tissue sections (50 um thick) from SS (bottom) and AA (top) mice. Areas of fluorescence indicate VCAM-1 deposition. These images were taken after spectral unmixing but prior to transferring to ImageJ for binarizing and overlaying masks. Adhesion factor expression was measured by analyzing fluorescence intensity and reported as relative fluorescence units (RFU) per millimeter squared of brain tissue. **(B)** Average area of VCAM-1 coverage per μm^2^ **(C)** VCAM-1 expression compared to controls. **(D)** Representative image of stained tissue sections (50 um thick) from SS (bottom) and AA (top) mice. Fluorophore of interest was isolated to show fluorescent areas representing P-selectin deposition along the microvasculature. Areas of fluorescence indicate P-selectin deposition. **(E)** Average area of P-selectin coverage per μm^2^. **(F)** microvascular P-selectin expression (RFU/mm^2^). **(G)** Leukocyte adherence (defined as lasting two seconds or more) per 100 μm length of vessel per minute was higher in sickle cell mice (*p* < 0.001) (AA: *n* = 5; SS: *n* = 7). Error bars are standard error of means (SEM). ***p ≤ 0.001; ****p ≤ 0.0001. Mean comparisons done using Welch's corrected t-test. Results here are only from the mice in the baseline group. For VCAM-1 analysis, AA = 184 brain slices and SS = 215 brain slices, while for P-selectin analysis, AA = 189 brain slices and SS = 165 brain slices. For leukocyte rolling, AA = average of ~194 vessel segments; SS = average of ~390 vessel segments.

To better understand the potential underlying factors responsible for the disturbed hemodynamics observed above, we performed immunohistochemical (IHC) analysis of the brains from the sickle cell and control mice, after completion of 2 Photon imaging. The IHC analysis focused mainly on VCAM-1 and P-selectin for reasons already mentioned in the background section (25, 27, 36, 37). Our analysis showed that sickle cell mice had significantly larger area of VCAM-1 coverage expressed per μm^2^ (*p* < 0.0001), as well as expression/deposition (intensity) measured in RFU/mm^2^ (*p* < 0.0001) in the cerebral microvasculature compared to controls at baseline (Figures 3A–C). Considering both the intensity and coverage parameters for expression, high intensity areas of fluorescence are not necessarily localized to one specific brain area. Similarly, and to large extent not surprising, we also noted that sickle cell mice had significantly larger area of P-selectin coverage per μm^2^ (*p* < 0.0001) and expression/deposition (intensity) measured in RFU/mm^2^ (*p* < 0.0001) in the cerebral microvasculature compared to controls (Figures 3D–F). Using images from 2 Photon microscopy and custom MATLAB scripts as already described, we quantified leukocyte adherence in the cortical microvasculature. The result indicates a significantly higher frequency of leukocyte adherence events in sickle cell mice (4.83 ± 0.57 /100μm/min vs. 2.26 ± 0.37 /100μm/min; *p* = 0.002) compared to controls at baseline (Figure 3G). This result is unsurprising given the well-established role of elevated P-selectin and VCAM-1 in as markers of endothelial activation as well as mediators of leukocytes rolling (37, 61–65).

### Microinfarct frequency and area in sickle cell mice compared to age matched controls at baseline

**Figure 4:**
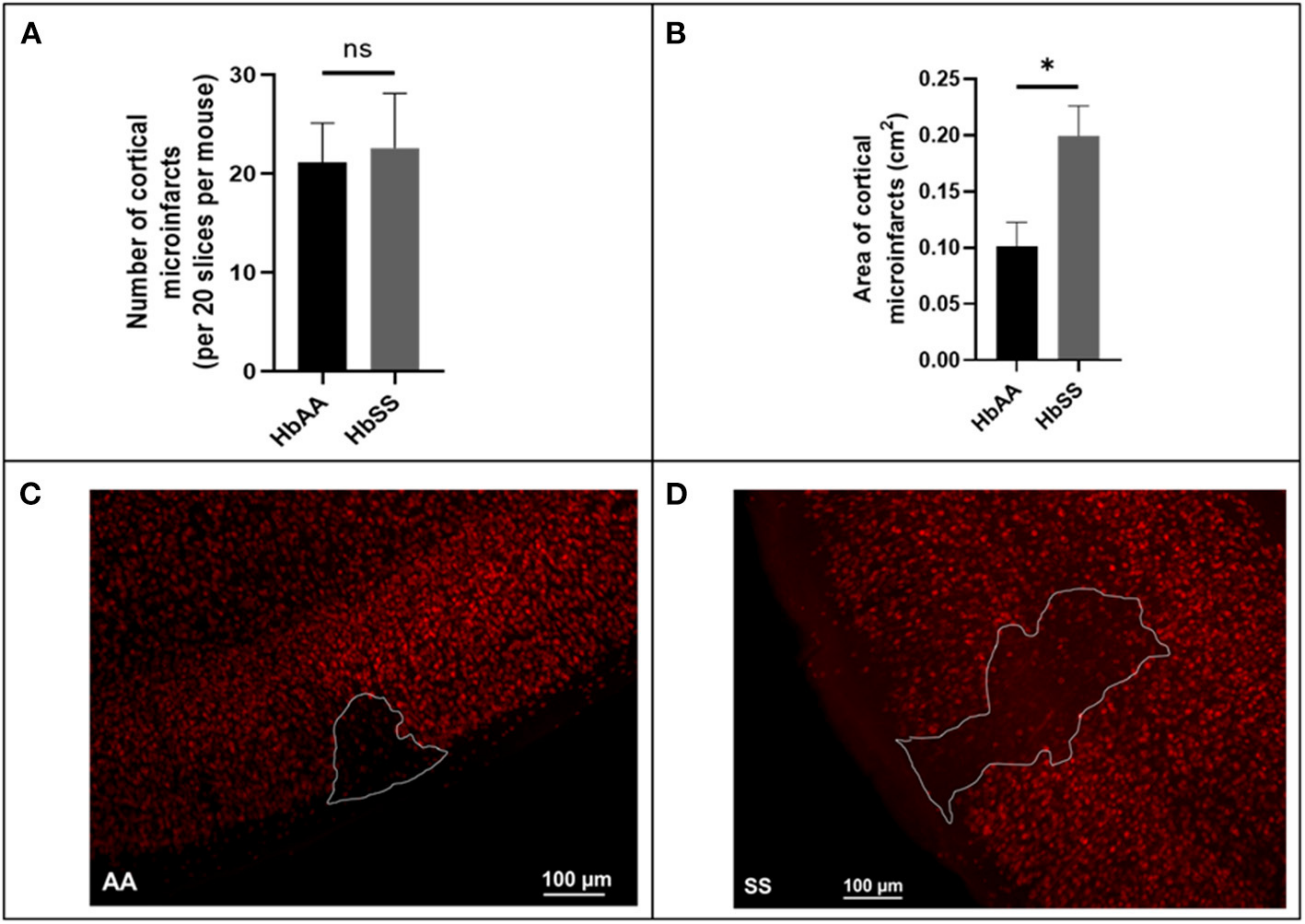
Baseline microinfarct data from 13-month-old mice. **(A)** Frequency of cortical infarcts per was not significantly different between HbAA and HbSS. **(B)** area of cortical infarcts in cm^2^. Error bars are standard error of means (SEM) (AA: *n* = 5; SS: *n* = 5). *p < 0.05. **(C)** Representative image of AA mouse microinfarct area. **(D)** Representative image of SS mouse microinfarct area. Mean comparisons done using Welch's corrected *t*-test.

Using a separate cohort of mice (male AA and SS mice that were 13 months old) that were neither transfused with packed red blood cells (pRBC) nor had cranial window implanted, we assessed the frequency and size (area) of microinfarcts (≥ 50 μM in diameter) in brain slices of sickle cell and control mice by examining all brain sections from sickle cell (*N* = 7) and control (*N* = 6) mice, encompassing ~20 sections per mouse spanning the entire cerebrum. Surprisingly, our analysis revealed that there was no significant difference in the frequency (22.60 ± 5.53 vs. 21.14 ± 4.0) for every 20 brain slices (Figure 4A). On the other hand, we noted that the cortical microinfarcts were significantly larger in sickle cell [0.2 ± 0.03 cm^2^ vs. 0.1 ± 0.02 cm^2^, *p* = 0.02] compared to the control mice (Figures 4B–D). This recapitulates data in our previous study (12).

### A single packed red blood cell transfusion improved cerebral microvascular hemodynamic measures in sickle cell mice

Given that blood transfusion therapy is still one of the most effective ways to prevent cerebrovascular complications such as stroke or recurrent stroke in SCD, (66–69) we decided to offer a single packed RBC transfusion to the sickle cell mice as described earlier. To accomplish this, we obtained whole blood from Townes humanized HbAA mice, spun it down, removed the plasma, resuspended the pellet in sterile cold PBS, centrifuged again (to wash), and then resuspended in sterile PBS at room temperature before proceeding immediately to transfuse. Sickle cell mice received 300 μL of packed RBC IV (with a goal of raising the hemoglobin by 1 g/dL), immediately following the pre-transfusion (PrT) 2 Photon microscopy session as described above. Control (HbAA) mice received 300 μL of normal saline about the same as the sickle cell mice. 2–3 weeks post transfusion, the mice underwent a second (post-transfusion) 2 Photon microscopy imaging. It is important to note, that while control mice received saline infusion, they did not receive blood transfusions and the PrT and PT designations are meant to indicate the fact that they were imaged at two time points that align with pre- and post-transfusion imaging time points in sickle cell mice.

**Figure 5:**
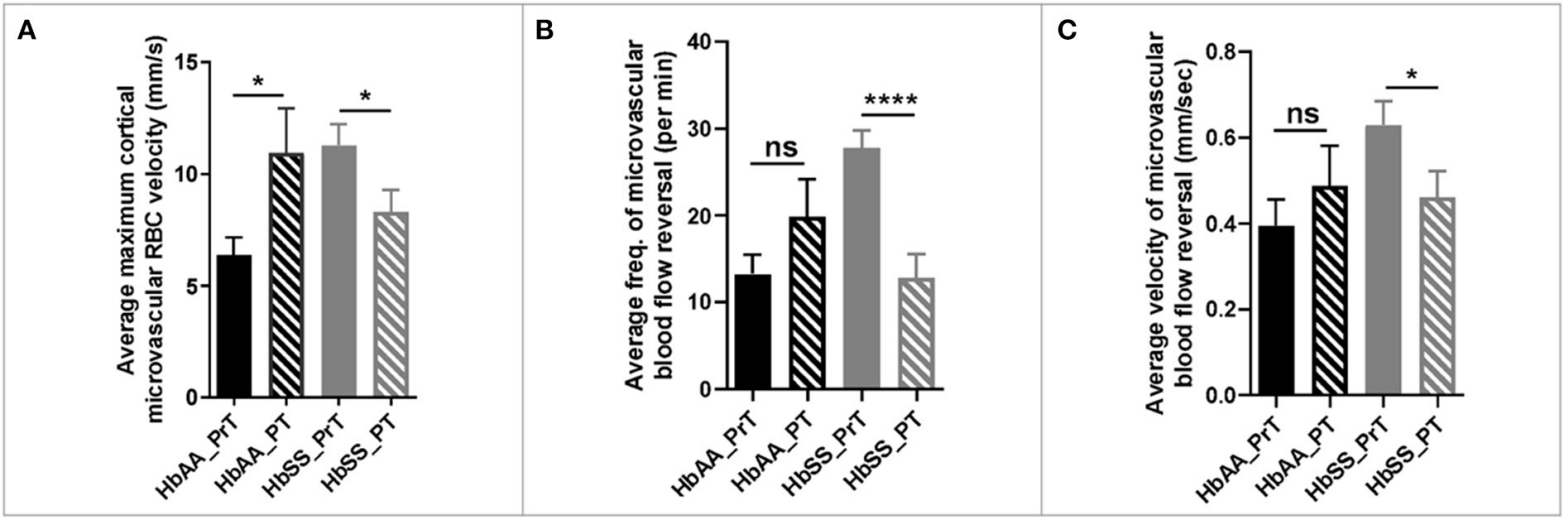
Hemodynamic analysis in sickle cell and control mice before and after blood or saline transfusion. **(A)** average maximum capillary RBC velocity in millimeters per second. **(B)** average frequency of capillary blood flow reversal per minute **(C)** average velocity of capillary blood flow reversal in millimeters per second (AA: *n* = 3–5; SS: *n* = 5–7). Error bars are standard error of means (SEM). NS, Not significant; *p ≤ 0.05; ****p ≤ 0.0001. Mean comparisons done using Welch's corrected *t*-test (HbAA_PrT was compared to HbAA_PT and HbSS_PrT was compared to HbSS_PT. There was no cross-genotype comparison since AA mice were not transfused with packed RBC. Prt, Pre-transfusion; PT, Post-transfusion. AA = average of ~73 vessel segments pre-transfusion and 23 vessel segments post-transfusion; SS = average of ~90 vessel segments pre-transfusion and 54 vessel segments post-transfusion.

The 2 photon microscopy images generated from the pre- and post-transfusion images were analyzed as described earlier. The result from our analysis showed that sickle cell mice that received packed RBC transfusion had a 36% (*p* = 0.03) reduction in maximum cortical microvascular RBC velocity, compared to their pre-transfusion values. On the other hand, we noted a 43% increase (*p* = 0.04) in maximum cortical microvascular RBC velocity in the control mice compared to their pre-transfusion values (Figure 5A). Also, compared to pre-transfusion levels, we noted significant lower frequency of microvascular blood flow reversal (Figure 4B, *p* < 0.0001) as well as decreased velocity (0.62 ± 0.06 mm/sec vs. 0.46 ± 0.06 mm/sec, *p* = 0.04) of cerebral microvascular blood flow reversal (Figure 4C) in sickle cell mice that were transfused with packed RBC. This suggests that blood transfusion treatment significantly improves hemodynamic abnormalities in sickle cell mice.

### Packed RBC transfusion decreases endothelial activation in cerebral microvasculature by decreasing expression/deposition of VCAM-1 and P-selectin, and leukocyte adherence in sickle cell mice

**Figure 6:**
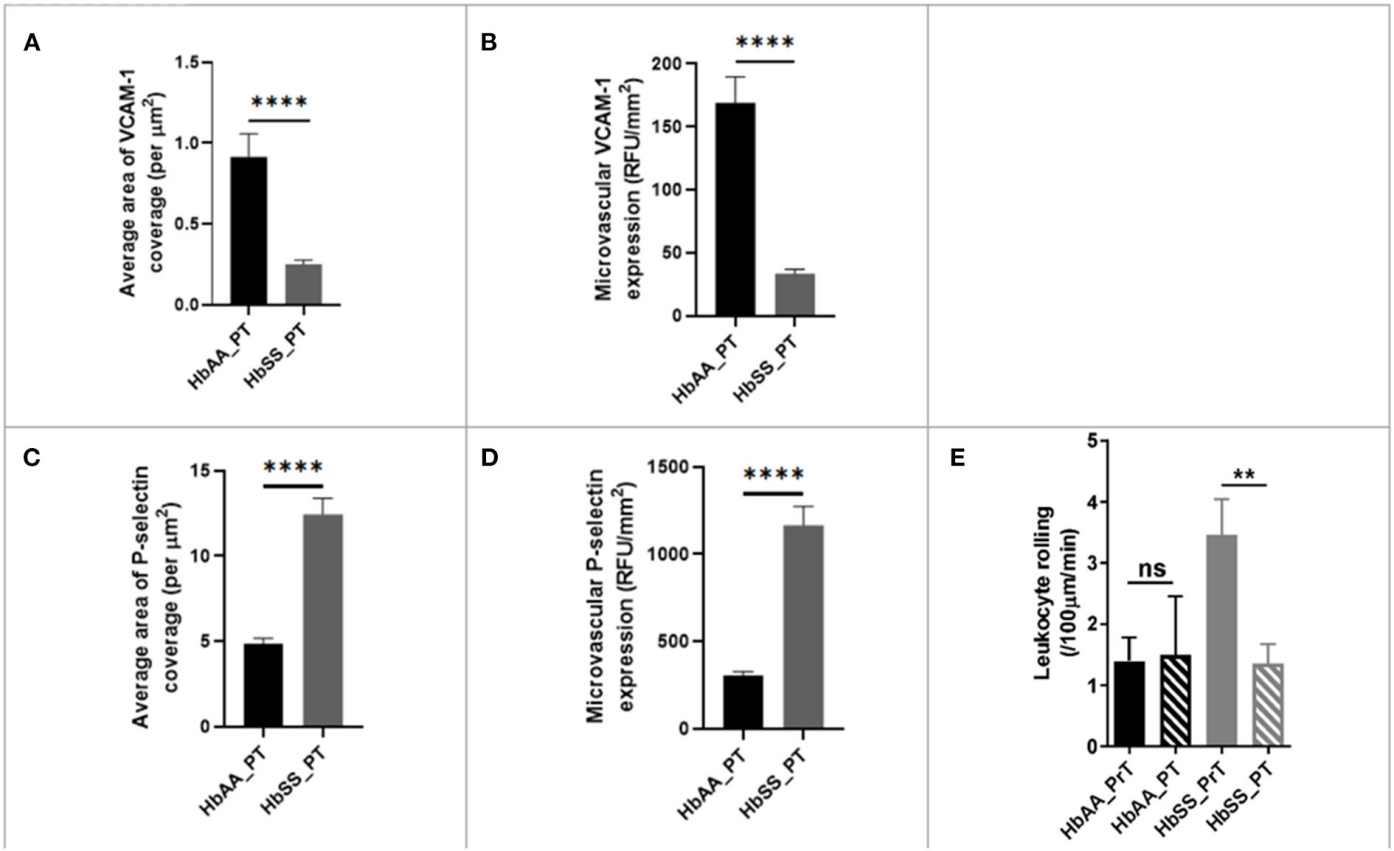
Blood transfusion improves cerebral vascular endothelium in sickle cell mice. **(A)** Average area of VCAM-1 coverage per μm^2^ in AA mice compared to SS. **(B)** Microvascular VCAM-1 expression in RFU/mm^2^ in AA mice compared to SS. **(C)** Average area of P-selectin coverage per μm^2^ (*p* < 0.0001). **(D)** Microvascular P-selectin expression (RFU/mm^2^). **(E)** Leukocyte adherence in AA and SS mice pre- and post-transfusion. AA: *n* = 3–5; SS: *n* = 5-7. Error bars are SEM. NS, Not significant; **p ≤ 0.01; ****p ≤ 0.0001. Mean comparisons done using Welch's corrected *t*-test. For VCAM-1 analysis, AA = 73 brain slices and SS = 147 brain slices, while for P-selectin analysis, AA = 162 brain slices and SS = 164 brain slices. For leukocyte rolling, AA = average of ~73 vessel segments pre-transfusion and 23 vessel segments post-transfusion; SS = average of ~90 vessel segments pre-transfusion and 54 vessel segments post-transfusion.

As stated earlier (Figure 3B), packed RBC transfusion is one of the mainstays for the prevention of stroke and neurovascular complications of SCD. Thus, as reported earlier, we used IHC to examine whether the single bolus of packed RBC transfusion had any impact on endothelial activation and therefore expression/deposition of VCAM-1 and/or P-selectin. Analysis of the IHC images, showed that sickle cell mice had significantly lower coverage (0.25 ± 0.02 /μm vs. 0.91 ± 0.14 /μm, *p* < 0.0001) compared with control mice. Also, when compared to the values obtained for sickle cell mice at baseline (Figure 3B), sickle cell mice that were transfused with packed RBC had a more than 7-fold lower VCAM-1 coverage. Additionally, microvascular VCAM-1 expression/deposition was significantly lower in sickle cell mice post transfusion (33.45 ± 3.44 RFU/mm^2^ vs. 168.90 ± 20.71 RFU/mm^2^, *p* < 0.0001) compared with control mice. And, when compared with values measured at baseline for sickle cell mice that were not transfused (Figure 3C), sickle cell mice that were transfused with packed RBC had an ~7-fold lower VCAM-1 expression/deposition in the cerebral microvasculature (Figures 6A,B). We also examined P-selectin coverage and expression/deposition as described earlier and observed that microvascular P-selectin coverage (12.45 ± 0.94 /μm vs. 4.85 ± 0.33 /μm, *p* < 0.0001) and expression/deposition (1165.00 ± 109.20 RFU/mm^2^ vs. 302.30 ± 24.87 RFU/mm^2^, *p* < 0.0001) were higher among sickle cell mice that were transfused with packed RBC, compared with controls (Figures 3C,D). Notwithstanding, when compared to values measured at baseline for sickle cell mice (Figures 3E,F), sickle cell mice that received packed RBC transfusion, had an approximately 3-fold (12.45 ± 0.94 /μm vs. 37.34 ± 2.16 /μm) and 2.4-fold (1165.00 ± 109.20 RFU/mm^2^ vs. 2742.00 ± 169.70 RFU/mm^2^) lower P-selectin coverage and expression/deposition respectively (Figures 6C,D). Finally, we examined pre- and post- packed RBC (for sickle cell mice) and saline (for controls) transfusion leukocyte adherence events. We noted that there was no significant difference in leukocyte adherence events between both time points for the control mice. However, sickle cell mice transfused with packed RBC had a significant reduction in leukocyte adherence events (1.35 ± 0.32 /100μm/min vs. 3.46 ± 0.58 /100μm/min; *p* = 0.0017) compared to pre-transfusion levels (Figure 6E). The data here suggests that a potential underlying benefit of transfusion for reduction of VOE could be *via* mitigating endothelial activation and therefore leukocyte adherence events.

## Discussion

Cerebrovascular abnormalities, including strokes and microinfarcts, have been well-documented in sickle cell disease and are associated with cognitive impairment (14, 70). In addition, high levels of adhesion factors have been implicated in SCD related complications, where they act as mediators of cellular (especially leukocyte) endothelial interactions and therefore vaso-occlusion (71, 72). Taken together, these events are crucial in the understanding of neurological pathology in SCD. The goal of this study was to examine the role of endothelial adhesion molecules (VCAM-1 and P-selectin) in cerebral microvascular hemodynamics, as well as the impact of blood transfusion treatment on these parameters (hemodynamics and expression of adhesion molecules). This study showed that compared to age-matched controls, aged Townes sickle cell mice have an abnormal cerebral microvascular hemodynamic profile, which is also associated with increased leukocyte adherence that seems to be mediated by a higher expression of adhesion factors (VCAM-1 and P-selectin). We also observed that these abnormalities were reduced, and in some instances, reversed, by blood transfusion treatment.

We observed a significantly higher average maximum microvascular RBC velocity in sickle cell mice compared to controls (Figure 2A). This finding corresponds with that from a previous study that showed higher capillary RBC velocity in aged sickle cell mice (12) as well as with observations in children and adults with SCD (25). The average maximum cortical blood flow velocity may be an indication of a compensatory mechanism for poor cerebral perfusion from downstream narrowing or obstruction and/or anemia. It is important to note that we are unable to precisely report capillary RBC velocity because we did not stain for smooth muscle actin, which would have enabled us to discriminate capillaries from pre-capillary arterioles and/or post capillary venules. Thus, our 2 Photon imaging may have included precapillary arterioles and post-capillary venules, and we refer to this throughout the paper as “microvasculature”. Notwithstanding, it is well known that besides the cortical capillaries, other segments of the cerebral vascular tree, such as the arteries and arteriole, carotid, and vertebral arteries, are affected in SCD, (73, 74) and the large vessel changes might reflect a later manifestation of microvascular abnormalities such as documented in this study. Furthermore, sickle cell mice had greater instances and higher velocity of blood flow reversal (Figures 2B,C). The exact mechanism for these reversals is unclear, however, studies (75, 76) suggest that experimental occlusion of either arterioles or venules results in cortical microvascular blood flow reversals as documented here. Thus, our finding suggests that the spontaneous blood flow reversals observed in our study could be due to spontaneous cerebral microvascular VOEs. While peripheral VOEs which are a hallmark of SCD and are well documented, cerebral microvascular VOEs have not been documented until recently (12) and are potentially manifesting in our study as blood flow reversals. These disturbances/turbulence in flow may also lead to endothelial activation. Overall, our report of a high velocity of flow, is corroborated by a recent report among patients with SCD, where using multiple-inflow-time arterial spin labeling, they showed a significantly higher cerebral blood flow in patients with SCD compared to controls (77).

Another important observation from our study is the elevated baseline expression/deposition of cerebral microvascular VCAM-1 and P-selectin in sickle cell mice compared to controls (Figures 3B–F). This data suggests that endothelial dysfunction may play a large role in SCD-related cerebral micro vasculopathy and thus neurovascular complications. The high maximum velocity and frequent flow reversals found in these mice may constitute some of the mechanical forces that trigger the expression of these endothelial adhesion factors (25, 78, 79). A recent study documented increased expression of VCAM-1 in the endothelium of aortic valve leaflets when they were exposed to shear stress (80). Thus, in concert with the prior stated mechanical damage to the endothelium, resulting from the physical contact with sickle RBC, these mechanical forces contribute to the promotion of microvascular thrombus generation from increased released of tissue factors, exposure of platelets to subendothelial tissues and therefore formation of platelet aggregates. This further promotes and increases the likelihood of cerebral microvascular VOEs and highlights the value of closely examining the role of endothelial adhesion factors in cerebral microvasculopathy (81).

Given the observations (Figures 3B–F), it is therefore no surprise for us to see a significantly higher frequency of leukocyte adherence in sickle cell mice compared to controls. This increased frequency of leukocyte adherence may also be associated with the higher expression of adhesion factors observed in Figures 3B–F. For example, VCAM-1 is the primary means by which leukocytes bind to the endothelium, using VLA-4, while P-selectin is one of the primarily means *via* which neutrophils interacts with the endothelium. Several studies in both humans and mice have shown that P-selectin and VCAM-1 are heavily implicated in SCD vascular dysfunction (26, 78, 82). These studies suggest that leukocyte-endothelial interactions (possibly mediated by VCAM-1 and/or P-selectin) are predominantly occurring in the post-capillary venules (83–86). Furthermore, recent clinical trials with crizanlizumab, a humanized P-selectin monoclonal antibody, have shown incredible promise, with patients with SCD who were treated with the drug, experiencing significantly fewer vaso-occlusive pain crises compared to patients on placebo, although these studies have no cerebral endpoints (38, 87). Another *in-vitro* study of Crizanlizumab showed inhibition of leukocyte adherence to P-selectin under physiologic blood flow conditions (44). Although the long-term benefits of crizanlizumab are yet to be determined, based on these previous studies and our results, it is tempting to predict that long-term use of crizanlizumab may attenuate some features of cerebral microvasculopathy and possibly SCD-related neurovascular pathologies, *via* reduction occurrence of VOEs in in the cerebral microvasculature (38, 44, 87).

Additionally, we noted that at baseline (without any intervention) the frequency of spontaneous microinfarct was not significantly different. The size (area) was significantly larger among sickle cell mice compared to controls (Figure 4B). The exact mechanism behind this is not yet clear. In a prior study, Luo et al. (88) reported that following middle cerebral artery occlusion, Townes sickle cell mice had significantly larger cerebral infarct size, compared to controls (88). Based on the rest of our data, it is plausible to reason that the growth of the infarct might be a self-propagating process (89) in the background of increased endothelial expression of adhesion molecules, increased leukocyte adherence and therefore higher frequency of VOEs as reported earlier in this section. This is a mechanism that warrants further investigation, and our lab is actively looking into this as it could also represent a therapeutic target for reducing the well described SCD-related cognitive decline which occurs in children and adults in the absence of an overt cerebral injury (90, 91).

Our observation of an apparent “normalization” of hemodynamic parameters/measured with packed RBC transfusion, compared to pre-transfusion levels in sickle cell mice and compared to controls (Figures 5A–C) was an intriguing albeit unsurprising finding. This result is exciting as it represents the first-time demonstration of a possible underlying mechanism of the benefits of blood transfusion. The dramatic change in maximum cerebral microvascular blood flow velocity could imply an improved ability to effectively perfuse the brain tissue without needing additional output velocity as a compensatory mechanism. According to this data, it seems that blood transfusion might have as a benefit, the normalization of cerebral microvascular dysfunction as a mechanism for the reported benefit in stroke prevention reported in children with SCD (48). Additionally, it is also possible that the reduction in flow velocity could be due to a decreased in VOEs, as we also observed a reduction in frequency (Figure 5B) and velocity (Figure 5B) of blood flow reversal. Furthermore, we also noted that compared to pre-transfusion or baseline levels, there was a significantly less microvascular expression and deposition of VCAM-1 and P-selectin, as well as significant decrease in leukocyte adherence in the cerebral of sickle cell mice. Taken together, the significant difference in expression and deposition of adhesion molecules combined with the reduction in leukocyte adherence, might account for some if not all the improvement seen in cerebral hemodynamic parameters. Due to the short duration of the blood transfusion therapy in addition to the fact that the assessment of cortical microinfarcts were performed post-mortem, we were unable to examine the impact of blood transfusion on frequency or size of the infarct as a function of the lower expression/deposition of adhesion endothelial adhesion molecules and/or leukocyte adherence. However, this will be the subject of future investigations in our laboratory.

A limitation of our study was that the expression of adhesion factors was quantified in the whole brain while hemodynamic changes were measured in cortical microvasculature. Nevertheless, our study corroborates the findings of several *in vivo* and *in vitro* studies by showing evidence of leukocyte-endothelial interactions, likely promoted by increased expression of adhesion factors expression. As mentioned earlier, we were also not able to reliably evaluate the impact of blood transfusion on frequency or size of cortical microinfarcts due to the short duration of time (2 weeks) from packed RBC transfusion to imaging and sacrifice of the mice. Future study designs have already worked out ways around this limitation using longitudinal approach to imaging. Due to equipment availability and other logistical reasons, we were also not able to access the post-transfusion hemoglobin levels and as such we are unable to make a firm statement with regards to how much the hemoglobin levels of the sickle cell mice went up post transfusion. Finally, we are not able to directly infer from our current data, a causal relationship between the improvement of hemodynamic parameters and lower expression of adhesion factors. However, ongoing studies in our lab using bone marrow chimera as well as sickle cell mice null for P-selectin and VCAM-1, should enable us to make such inference in the future.

## Conclusion

By examining hemodynamics and adhesion factors using two-photon laser microscopy and post-mortem immunohistochemistry in both pre- and post- transfusion sickle cell mice, we were able to document evidence of cerebral microvasculopathy in sickle cell mice. Additionally, we were able to show that blood transfusion might exert its benefit of preventing neurovascular complications by mitigating cerebral microvascular endothelial activation and the underlying mechanism of VOEs. Thus, our study potentially highlights one of the mechanisms that may be contributing to how blood transfusion prevents stroke and other neurovascular pathologies. The significant decrease in VCAM-1 and P-selectin expression in the brain following blood transfusion offers a particular new avenue for investigation, as well as therapeutic targets.

## Data availability statement

The raw data supporting the conclusions of this article will be made available by the authors, without undue reservation.

## Ethics statement

The animal study was reviewed and approved by Institutional Animal Care and Use Committees (IACUC) of Emory University and the Medical University of South Carolina.

## Author contributions

HH designed the experiment and performed the final critical review. NA, DA, JJ, MS, and HH performed experiments and data analysis. NA, OTG, and HH wrote the manuscript. DA, JJ, and MS provided critical review. All authors endorsed the submission of this manuscript.
